# Large-scale lagovirus disease outbreaks in European brown hares (*Lepus europaeus*) in France caused by RHDV2 strains spatially shared with rabbits (*Oryctolagus cuniculus*)

**DOI:** 10.1186/s13567-017-0473-y

**Published:** 2017-10-28

**Authors:** Ghislaine Le Gall-Reculé, Evelyne Lemaitre, Stéphane Bertagnoli, Céline Hubert, Sokunthea Top, Anouk Decors, Stéphane Marchandeau, Jean-Sébastien Guitton

**Affiliations:** 1 Avian and Rabbit Virology, Immunology and Parasitology Unit, Anses, French Agency for Food, Environmental and Occupational Health & Safety, Ploufragan-Plouzané Laboratory, BP 53, 22440 Ploufragan, France; 2 University Bretagne Loire, CS 54417, 35044 Rennes Cedex, France; 3 IHAP, University of Toulouse, INRA, ENVT, 31076 Toulouse, France; 4 Research Department, ONCFS, National Hunting and Wildlife Agency, USF, BP 20, 78610 Le Perray-en-Yvelines, France; 5 Research Department, ONCFS, National Hunting and Wildlife Agency, UFP, CS 42355, 44323 Nantes Cedex 3, France

## Abstract

Rabbit haemorrhagic disease virus (RHDV) is a lagovirus that causes rabbit haemorrhagic disease (RHD) in European rabbits (*Oryctolagus cuniculus*). In 2010, a new genotype called RHDV2 emerged in France. It exhibits a larger host range than classical RHDV strains by sporadically infecting different hare species, including the European hare (*Lepus europaeus*). Phylogenetic analyses revealed that closely related RHDV2 strains circulate locally in both hares and rabbits, and therefore that RHDV2 strains infecting hares do not belong to a lineage that has evolved only in this species. We showed that RHDV2 is widely distributed in France and that it was responsible for more than a third of cases of lagovirus disease in European hare populations in 2015. The oldest RHDV2 positive hare was sampled in November 2013 and we reported two hares co-infected by EBHSV and RHDV2. All together, our results raise important epidemiological and evolutionary issues. In particular, along with the potential emergence of recombinant EBHSV/RHDV2 strains in hares, the enlargement of the host range changes the host population structure of RHDV2 and may alter the impact of the virus on rabbit and hare populations.

**Electronic supplementary material:**

The online version of this article (doi:10.1186/s13567-017-0473-y) contains supplementary material, which is available to authorized users.

## Introduction

Rabbit haemorrhagic disease (RHD) is a highly infectious and often fatal viral disease of European rabbits (*Oryctolagus cuniculus*). The disease was first reported in China in 1984 and subsequently in many countries throughout the world [1]. The aetiological agent is a non-enveloped single-stranded positive-sense RNA virus belonging to the genus *Lagovirus* of the family *Caliciviridae* and called rabbit haemorrhagic disease virus (RHDV). RHD induces high mortality in both domestic and wild rabbit populations. Clinical signs and histopathological characteristics include severe necrotizing hepatitis and massive disseminated intravascular coagulation [2].

Until 2010, phylogenetic analyses revealed the progressive emergence of distinct but closely related variants of RHDV [3–5]. In 2010, a new genotype of RHDV, named RHDV2, was identified in France with distinct pathogenic, genetic and antigenic profiles [6, 7]. RHDV2 rapidly spread throughout Europe, causing significant losses in both domestic and wild rabbits [6, 8–11], and was then detected in different islands, in Australia, in Northern America and in Africa [12–18]. In Western Europe, RHDV2 is responsible for almost all cases of RHD in both domestic and wild rabbit, cases of RHD caused by former RHDV strains being rare [6, 9, 13, 19, 20]. RHDV2 quickly evolved and recombination with rabbit lagoviruses that co-circulate in Europe has been evidenced in the Iberian Peninsula [21, 22]. These recombinations include the region of the genome encoding the structural VP60 and VP10 proteins of RHDV2 with the upstream region of the genome encoding the non-structural proteins, either from the RHDV genogroup G1 or from non-pathogenic lagoviruses.

RHDV2 exhibits a broader host range than classical RHDV by infecting not only rabbits but also different hare species (*Lepus capensis mediterraneus*, *Lepus corsicanus*, *Lepus europaeus*, *Lepus timidus*) [23–27]. RHDV2-infected hares show very similar clinical signs to those induced by European brown hare syndrome (EBHS): hyperaemic trachea sometimes containing uncoagulated blood, hepatitis necrosis, splenomegaly and congestion of the other organs and tissues [23, 25, 26]. EBHS was first described in 1980 [28] and is caused by another lagovirus called EBHSV, which is known to infect and cause disease in several hares species (*L. europaeus*, *L. timidus* and *L. corsicanus*). In the European hare (*L. europaeus*), some cases of hares showing lesions similar to EBHS (EBHS-like disease) caused by RHDV2 were reported in Italy in 2012, in Spain in 2014 and in Australia in 2016 [24, 26]. To date it is unclear whether RHDV2 may cause outbreaks in hare populations. Indeed, Velarde et al. [26] described sporadic cases in Italy (one hare) and Spain (two hares) and the data recorded in Australia do not indicate whether the five positive hares correspond to outbreaks or to spillover events [24].

Assessing the spreading level of RHDV2 into hare species, and especially *L. europaeus*, is an important issue because it may have significant epidemiological and evolutionary implications in areas where European hares are widespread like France for instance. For example, a large-scale spread into hare populations would increase the probability of recombination events between RHDV2 and EBHSV. It would also change the spatial structure of RHDV2 host population, which is known to influence the evolution of pathogens [29, 30]. Yet rabbit populations are patchily distributed [31, 32], while hare populations are more continuously distributed [33].

Therefore, the present study aims at detecting RHDV2 in European hares which died of lagovirus disease in France and at assessing the relative proportion of cases due to EBHSV and RHDV2. We searched for RHDV2 in a sample of 50 dead hares collected between 2013 and early 2015 and compared these strains to those involved in RHD outbreaks in rabbits during the same period. We then carried out a large scale molecular epidemiological study to estimate the proportion of lagovirus disease caused by RHDV2 in 2015 in France. This latter analysis was performed on 208 liver samples taken on hares that died from an EBHS-like disease in 2015.

## Materials and methods

### Detection and molecular characterization of RHDV2 in hares

#### European hare samples

We randomly sampled 50 European hares collected by the SAGIR network between January 2013 and February 2015 displaying lesions compatible with EBHS at necropsy (Table 1). The livers were stored at −20 °C before being sent to the Anses’ laboratory for further molecular analyses.

**Table 1:** **Summary of samples, methods and results on hares**

Objective	Sample size	Collection period	Methods used	Virus strains
Detection and molecular characterization of RHDV2 in hares	50^a^	01/2013 to 02/2015	RT-PCRs U38/EBHS9 and VP60, search for recombinant strains (RT-PCR 1U/1L), sequencing	7 RHDV2 (of which 3 recombinant strains and 1 coinfection RHDV2/EBHSV)
Relative proportion of RHDV2 and EBHSV in lagovirus infections	208^a^	2015	rRT-PCR RHDV2 and rRT-PCR EBHSV	32 RHDV2, 54 EBHSV and 1 coinfection RHDV2/EBHSV

Complete information on the lagovirus positive samples is reported in the Additional files 1 and 2.

^a^Ten samples have been used for both objectives.

SAGIR is a generalist incident-based surveillance network for epidemiological vigilance towards wildlife diseases dealing with early detection and early warning [34]. The vigilance relies on a diagnostic process, based on a transdisciplinary approach (epidemiology, ecology, toxicology and pathology). A gross pathologic examination is performed on each submitted animal. According to field and epidemiological clues as well as a gross pathologic and clinical picture, additional tests (parasitology, bacteriology, virology, histology) helpful to investigate the aetiology of death are prioritized. Calicivirus investigation relies therefore on epidemiological or/and ongoing process type (acute) or/and evocative lesion.

#### Search for lagoviruses by RT-PCR-sequencing

Each frozen liver sample was thawed and total RNA was extracted from 100 µL of the obtained exudate using the NucleoSpin^®^ RNA kit (Macherey–Nagel) according to the manufacturer’s instructions. RNAs were analyzed to obtain molecular data using the RT-PCR U38/EBHS9 [35] that can amplify a part of the capsid protein gene of both EBHSV and RHDV/RHDV2. Reverse transcriptions were performed using oligo(dT) as primers (Invitrogen) and SuperScript™ II Reverse Transcriptase (Invitrogen). The cDNA were amplified using the AmpliTaq Gold DNA polymerase (Applera Applied Biosystems) (10 min at 94 °C, followed by 30 cycles at 94 °C for 30 s, 57.6 °C for 30 s and 72 °C for 45 s, then 10 min at 72 °C) and visualized by electrophoresis on agarose gel.

When they existed, PCR products were purified using MinElute™ PCR Purification Kit (QIAGEN). DNA sequences were determined in both directions using the PCR primers and Big Dye Terminator v3.1 (Life Technologies) as recommended by the manufacturer, and analyzed with an “ABI Prism 3130 Genetic Analyzer” (Applied Biosystems). Nucleotide sequences were compiled using Vector NTI Advance 11.5 (Invitrogen). In order to genotype the viruses, the sequences were aligned against the nucleotide sequences present in databases using the standard nucleotide BLAST (blastn) program available in the National Center for Biotechnology Information (NCBI) web BLAST service.

#### Amplification and sequencing of the capsid protein genes of RHDV2

The complete sequence of the gene encoding the capsid protein of seven RHDV2 were obtained following specific full-length gene PCR amplification using the Expand™ High Fidelity enzyme (Roche Applied Science) and the primers 12U and 15L [6]. For one RHDV2 (E14-73), the capsid protein gene amplification was obtained using the primers 12U2 (5′-GGATCGTCTCGGTAGTACC, 5263–5282) and Calici7255-Rev (5′-CTACACTAGCATCATTATGCAT, 7268–7247). The cycle conditions were those indicated by the manufacturer, with an annealing temperature of 56.8 and 54 °C, respectively, during 30 s.

The amplified DNA were purified and sequenced as mentioned above using the PCR primers as well as several primers designed in the inner region of the templates (primers sequences available upon request). The sequences were compiled using Vector NTI Advance 11.5 (Invitrogen).

#### Phylogenetic analysis

Phylogenetic relationships were inferred using the VP60 gene sequences of seven RHDV2 characterized in brown hares and rabbit lagovirus VP60 sequences available in databases. We also included the VP60 gene sequences of nine French RHDV2 circulating in wild rabbits and farms between January 2013 and November 2014 (Additional file 1). The sequence of the French reference EBHSV strain EBHSV-GD (Z69620) was used as an outgroup to root the trees. Phylogenetic analyses were conducted in MEGA software version 5 [36]. The neighbor-joining method was implemented with the pairwise deletion option and based on the Kimura 2-parameter model including transition and transversion substitutions. Codon positions included were 1st + 2nd + 3rd. Search for the trees was implemented with the close-neighbor-interchange algorithm. Reliability of the trees was assessed by bootstrap with 1000 replicates. Pairwise nucleotide distance comparisons based on the *p*-distance model were conducted in MEGA5.

#### Nucleotide VP60 sequence accession numbers

Nucleotide VP60 sequences of six RHDV2 characterized in French hares (E13-37, E14-03, E14-40, E14-45, E14-115 and E15-12) and nine RHDV2 characterized in French rabbits (13–22, 13–69, 13–71, 13–100, 13–122, 13–165, 14–02, 14–59 and 14–114) are available in databases under the EMBL/GenBank Accession Numbers LT168833 to LT168847. The RHDV2 E14-73 VP60 sequence is available in databases under the Accession Number LT549473.

#### Search for RHDV2 recombinant strains

To determine whether the characterized RHDV2 strains were recombinant or not with RHDV G1 or RCV-A1-like viruses as are some RHDV2 from the Iberian Peninsula [21], we genotyped a 518 bp-long region of the genome upstream of the VP60 gene and close to the 5′ end of the genome (nt 13 to nt 530). This region presents a low sequence similarity between the different rabbit lagovirus genotypes (RHDV, RHDV2, RCV-A1 and RCV-like non-pathogenic lagoviruses) which enables them to be distinguished. Even if the sequenced region does not cover the main recombination breakpoints identified in a region close to the cleavage site of the capsid gene [21, 37], this PCR is sufficient to detect recombinant RHDV2 strains in hares. Indeed, since there is no evidence of European hare sensibility to RHDV G1 or RCV-A1-like viruses, positive results cannot be interpreted as the detection of a co-infection. Amplification was carried out using AmpliTaq Gold DNA polymerase (Applera Applied Biosystems) (10 min at 94 °C, followed by 30 cycles at 94 °C for 30 s, 54 °C for 30 s and 72 °C for 45 s, then 10 min at 72 °C) and the primers 1U (5′-GATTAGGCCGTGAAARTTATG, 1–12) and 1L (5′-CAACGTCAACAAACTTGTCC, 550–531). The amplified DNA were purified and sequenced as mentioned above using the PCR primers. The sequences were compiled using Vector NTI Advance 11.5 (Invitrogen) and genotyping as previously described.

### Relative proportion of RHDV2 and EBHSV in lagovirus infections in 2015

#### European hare samples

Among the European hares collected throughout France in 2015 by the SAGIR network displaying lesions compatible with EBHS at necropsy, we sampled the 208 hares for which a virological diagnostic has been performed. Ten of those 208 samples also belong to the 50 samples that have been previously analysed for detection and molecular characterization of RHDV2 in hares (Table 1). The livers were stored at −20 °C before being sent to the Anses’ laboratory for further molecular analyses.

#### EBHSV and RHDV2 screening by real-time RT-PCRs

Each frozen liver sample was thawed and total RNA was extracted from 100 µL of the obtained exudate using the NucleoSpin^®^ RNA (individual column) or NucleoSpin^®^ 8 RNA (8-well strip) kits (Macherey–Nagel) according to the manufacturer’s instructions.

The rRT-PCR EBHSV developed to amplify the target gene and the internal control (Intype IC-RNA, QIAGEN) in a single tube was performed using the thermal cycler “7500 Real Time PRC System” (Applied Biosystems). Amplification was carried out using the QuantiTect Probe RT-PCR Kit (QIAGEN) according to the manufacturer’s instructions. Each reaction contained 5 µL of extracted RNA template and a final concentration of 100 nM of probe EBHS-Pro2 (6-FAM 5′-CTGACGCCCCTGGCACCGCTA BHQ-1^®^, 5302–5322) and IC-Probe (Cy 5^®^ 5′-AGCACCCAGTCCGCCCTGAGCA BHQ-2^®^), of 400 nM of the target gene primers EBHS-6Fwd (5′-AATGTTATGGAGGGTAAGCC, 5277–5296) and EBHS + 115Rev (5′-GATGCTATAACGTTGTCAGC, 5416–5397), and of 400 nM of IC primers IC-1F (5′-GACCACTACCAGCAGAACAC) and IC-1R (5′-GAACTCCAGCAGGACCATG). Thermal cycling conditions used were 30 min at 48 °C, 10 min at 95 °C, followed by 40 cycles of amplification (95 °C for 15 s, 60 °C for 1 min). PCR results were analyzed using the 7500 Software (Applied Biosystems).

The rRT-PCR RHDV2 was performed on the same machine as described above by using the Power SYBR^®^Green RNA-to-CT™ 1-step kit (Applied Biosystems) according to the manufacturer’s instructions. Each reaction contained 5 µL of extracted RNA template and a final concentration of 200 nM of primers RHDV2S-F (5′-CGACAACAGATGGAATGGTG, 6063–6082) and RHDV2S-R (5′-AACCATCTGGAGCAATTTGG, 6310–6291). Thermal cycling conditions used were 30 min at 48 °C, 10 min at 95 °C, followed by 40 cycles of amplification (95 °C for 15 s, 60 °C for 1 min) finished with a default melting curve. Analyses of cycle threshold (Ct) and melting temperature (Tm) were calculated using 7500 Software (Applied Biosystems).

Doubtful results (Ct values close to the threshold) or results suggesting an EBHSV-RHDV2 co-infection were analysed again by RT-PCR-sequencing in order to obtain genotyping data.

## Results

### RHDV2 isolated in hares collected between 2013 and early 2015

**Figure 1:**
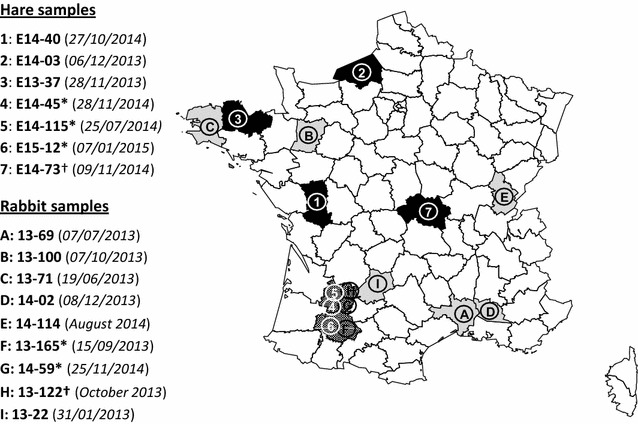
**Spatial distribution of analyzed RHD2 outbreaks in French hare and rabbit populations (January 2013–February 2015).** Data are pooled by department (administrative area) and the color indicates the species involved in the analyzed outbreaks. Black: *Lepus europaeus*; light grey: *Oryctolagus cuniculus*; dark grey: the two leporid species. White: no data. The numbers refer to RHDV2 strains characterized in hares and the letters to RHDV2 strains characterized in rabbits. The order of the numbers and the letters corresponds to the order in which the VP60 sequences are positioned in the phylogenetic tree from top to bottom (Figure 2). *Recombinant RHDV-G1/RHDV2 strains, ^†^unsuccessful 1U/1L PCR amplification.

The genotyping results obtained by RT-PCR-sequencing U38-EBHS9 performed on 50 hare samples collected between January 2013 and February 2015 revealed the presence of RHDV2 in seven samples (E13-37, E14-03, E14-40, E14-45, E14-73, E14-115, and E15-12) (Table 1). The oldest sample displaying RHDV2 infection has been collected in November 2013. These seven samples have been collected in different hare populations throughout France (Figure 1). In the E14-40 sample, our analysis showed a co-infection by both RHDV2 and EBHSV.

### Genetic and spatial relationships between RHDV2 strains characterized in hares and rabbits showed a strong epidemiological link

**Figure 2:**
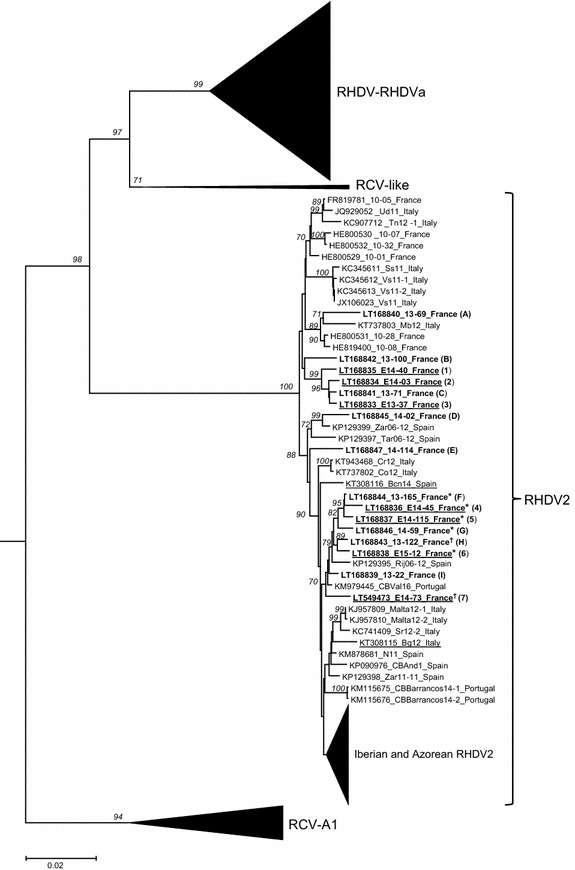
**Phylogenetic tree derived for VP60 nucleotide sequences of 381 rabbit lagoviruses including 15 French RHDV2.** The tree was obtained using the Neighbor-Joining method and was drawn to a scale of nucleotide substitutions per site. The percentages greater than 70% of replicate trees in which the associated taxa clustered together in the bootstrap test (1000 replicates) are given in italics before each major branch node. The European brown hare syndrome virus (EBHSV) strain GD (Z69620) was used as an outgroup to root the tree. The RHDV2 strains characterized in hares are underlined. The 15 French RHDV2 strains characterized in this study (in rabbits and hares) are in bold and their names are followed by the number (for hares) or the letter (for rabbits) mentioned in the Figure 1 for each strain. The RHDV, the non-pathogenic and weakly pathogenic lagoviruses, most of the Iberian and Azorean RHDV2 and the Australian non-pathogenic viruses were collapsed to lighten the figure. *Recombinant RHDV-G1/RHDV2 strains, ^†^unsuccessful 1U/1L PCR amplification.

We obtained the complete VP60 sequences of the seven RHDV2 strains isolated in hares. Phylogenetic analyses showed that these sequences clustered into the RHDV2 phylogenetic group and thus confirmed that the viruses isolated in hares are RHDV2 (Figure 2). These RHDV2 sequences are closely related to each other but distinct. They significantly clustered into two groups, the first one (E14-40, E14-03 and E13-37) showing an average nucleotide identity of 96.3% with the second one (E14-45, E14-115, E15-12 and E14-73). The two groups contained both hare and rabbit viruses, including several RHDV2 characterized in rabbits during the same period of time and closely related to RHDV2 characterized in hares (99–99.2% nucleotide identity). They also revealed a spatial structure, with a first group that gathered viruses circulating in north-western France and a second group that gathered RHDV2 from two nearby departments in south-western France (Figure 1).

### Search for recombinant strains

We identified recombinant strains in the south-western group only (Figure 1). The 1U/1L sequences of five RHDV2 strains, three isolated in hares (E14-45, E14-115 and E15-12) and two isolated in rabbits (13–165 and 14–59) showed ~ 98% nucleotide identity with Iberian RHDV-G1/RHDV2 recombinant strain sequences, suggesting a similar genetic origin.

### The molecular epidemiological survey revealed a high proportion and a large geographical distribution of lagovirus disease due to RHDV2 in 2015

**Figure 3:**
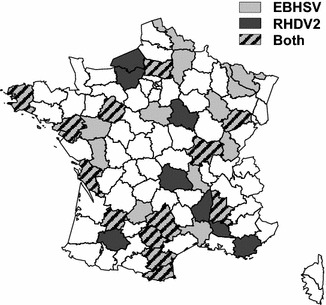
**Spatial distributions of EBHSV and RHDV2 outbreaks in hare populations in France in 2015.** Data are pooled by department (administrative area) and the color indicates the lagoviruses involved in the outbreaks. Department in white: negative samples for lagovirus or absence of sampling.

Prevalence of EBHSV and RHDV2 in hares that died of an EBHS-like disease in 2015 was estimated by analyzing 208 hare samples both by rRT-PCR EBHSV and rRT-PCR RHDV2. We showed that 42% (87/208) were infected by a lagovirus. Among them, 62% (54/87) were infected by EBHSV, 37% (32/87) by RHDV2, and 1% (1/87) co-infected by both viruses (Table 1). This survey also showed that EBHSV and RHDV2 were present in hare populations distributed throughout France (Figure 3). In addition, SAGIR records showed that at least three dead hares (E14-03, E14-45 and E15-12) were collected during severe outbreaks with several dead hares found in a restricted area. However, as this information was not systematically recorded, the outbreak severity of the four other cases remains uncertain.

## Discussion

Our study confirms the sensitivity of the European hare to RHDV2 and shows that closely related strains circulate locally in both rabbit and hare populations. It also demonstrates that RHDV2 is responsible for a large proportion of lagovirus disease cases in hare populations in France. These findings have several implications in terms of lagoviruses evolution and diagnosis strategy.

Our study confirms (i) the susceptibility of European hares to RHDV2 and (ii) the fact that RHDV2 and EBHSV cause similar diseases in this species, as previously observed [24, 26]. In addition, our data show that RHDV2 may cause severe outbreaks in European hares, which had not been previously reported [24, 26]. RHDV2 is therefore highly virulent in at least three lagomorph species, the European rabbit (*O. cuniculus*), the European hare (*L. europaeus*) and the Cape hare (*L. capensis mediterraneus*), and is probably less virulent in the Italian hare (*L. corsicanus*) [7, 23, 25]. Even though one case of RHDV2 infection has recently been reported in the Mountain hare (*L. timidus*), its virulence in this species remains undocumented [27].

Our results also provide new insights into RHDV2 epidemiology in hares since they demonstrate that RHDV2 strains infecting hares do not belong to a lineage that has evolved only in hares from a single species barrier crossing. Indeed, phylogenetic analyses show that RHDV2 strains characterized in hares do not cluster into specific branches but belong to several lineages including also strains isolated in rabbits: their genetic diversity shows that RHDV2 outbreaks in hares correspond to independent infections. On the other hand, their phylogenetic position within RHDV2 strains isolated in rabbits at the same period and in the same geographical areas suggests a strong epidemiological link between cases in rabbits and hares. This finding is strengthened by the characteristics of the strains isolated in south-western France in both species. Indeed, in this area, recombinant RHDV-G1/RHDV2 strains in rabbits and hares could be isolated. Altogether, these two features support the hypothesis of a local sharing of strains between the two species.

Another major finding is that hare infection by RHDV2 is common. Indeed, RHDV2 was involved in nearly 40% of cases of lagovirus disease in hares recorded in France in 2015 and was spatially widespread throughout France. This high proportion of RHDV2 in lagovirus disease cases may be due to intra-specific transmission among hares and also to inter-specific transmission from rabbits to hares, made possible by both the wide distribution of rabbits in France and the large spatial distribution of RHDV2 among rabbit populations.

The first case described in our study occurred in November 2013, which is later than the first case recorded in Italy in November 2012 [26]. However, since crossing the species barrier appears to be frequent, one cannot exclude that some hare infection by RHDV2 in France occurred earlier than 2013, shortly after its emergence in rabbits in 2010 [6]. Such a short time laps between RHDV2 detection in rabbits and hares was observed in Sardinia where RHDV2 was detected in Cape hares (*L. capensis mediterraneus*) only 2 months after the first case recorded in rabbits [25]. A large-scale survey on older samples is currently in progress to assess more precisely the date of the first crossing of species barrier in France.

Recombination is a common feature of rabbit lagoviruses, increasing their genetic diversity and driving their evolution [21, 37–39]. The sharing of the same virus by rabbits and hares may have major evolutionary consequences since it increases the number of viruses possibly involved and the number of hosts in which these recombination events may occur. This hypothesis is supported by the detection of two hares co-infected by EBHSV and RHDV2, which makes possible the recombination between these viruses previously considered to be specific to hares and rabbits, respectively.

The global host population size, but also its spatial structure, strongly influence the evolution of pathogens virulence and their demographic impact [29, 30]. The jump of RHDV2 to the European hare increases the connectivity between host populations because rabbit populations are patchily distributed while hares display a more continuous spatial distribution. Thus, the enlargement of host range may have contrasted effects on RHDV2 virulence and impact on rabbit and hare populations. First, a less fragmented host population could enable a steady circulation of the virus in both hosts and maintain higher immunity levels [40]. By increasing the herd immunity, the change of host range may therefore lead to a decrease of RHDV2 impact in rabbits. Conversely, the change in the spatial structure of the global host population may contribute to increasing the mean virulence of RHDV2. Indeed, Fouchet et al. [41], by modelling, suggest that “the mean virulence of RHDV increases with connectivity”. One may therefore expect a higher virulence of RHDV2, which is supported by recent data [42].

This also raises the question of the competition between three viruses (RHDV, RHDV2 and EBHSV) two of which are genus specific and one is able to infect both rabbit and hare species. Yet, RHDV2 is replacing RHDV in rabbit populations in south-western Europe [6, 9, 13, 19, 20]. Thus one may wonder about a possible future replacement of EBHSV by RHDV2 in hare populations. In this context, the role, evolution and spreading of recombinant strains, such as RHDV-G1/RHDV2, may also be of particular interest to assess whether recombinant strains benefit from a selective advantage.

Another consequence of RHDV2 infection in hares concerns the diagnostic strategy which has to be redefined in order to look for both EBHSV and RHDV2 in case of EBHS suspicion. A new rRT-PCR has been recently developed to amplify EBHSV and RHDV2 genes along with the exogenous internal control in a single tube (Lemaitre et al., unpublished data). This new method will allow the monitoring of the circulation of the two viruses in hares during epidemiological surveys and to detect the possible replacement of EBHSV by RHDV2. Moreover, in the case of surveys aiming at identifying the viral strains involved in lagovirus disease outbreaks, the detection of recombinant strains would also be useful for a better understanding of RHDV2 evolution in this species. Regardless of lagovirus strains identification, our data show that a high proportion (121/208 in 2015) of hares displaying lesions compatible with EBHS have not died of a lagovirus disease. Other causes may induce similar or resembling lesions and a better diagnosis of those cases will be another important issue for the future.

Lastly, RHDV2 ability to infect a wide range of species hosts and to cause in European hare a disease similar to EBHS should lead one to reconsider both viruses and diseases taxonomy. Until now, EBHSV and RHDV have been considered as two different virus species mainly on the basis of their distinct host range, hares and rabbits, respectively. The jump of RHDV2 to different hare species makes this classification obsolete. A new nomenclature based on genetic criteria and therefore more powerful has recently been proposed [43]. Similarly, the names of the diseases, EBHS and RHD, are related to the affected host. The passage of RHDV2 from the rabbit to the hare, in which it causes a disease in all respects similar to EBHS, shows the limits of this nomenclature. We are now dealing with a disease—EBHS—whose name calls back to a virus that is not always responsible for this disease. We therefore propose to rename this disease hare haemorrhagic disease (HHD) or hare lagovirus disease (HLV), independently of the viral strains that cause it. This nomenclature is similar to that adopted in the rabbit where RHD designates the disease, irrespective of the virus responsible for it, RHDV or RHDV2.

## Additional files

**Additional file 1.** **Lagovirus positive samples used for the detection and molecular characterization of RHDV2 in hares.** The table gives the sample number, the year of collection, the French department number, the genotype and the EMBL/Genbank accession number in nucleotide databases of the RHDV2 VP60 sequence of the lagoviruses characterized in hares (*n* = 7) and of nine RHDV2 characterized in dead rabbits during the same period of time. *Recombinant RHDV-G1/RHDV2 strains.

**Additional file 2.** **Lagovirus positive samples used for estimating the proportion of RHDV2 in lagovirus infection in hares.** The table gives the sample number, the year of collection, the French department number and the genotype of the lagoviruses characterized during the screening performed to estimate the relative proportion of RHDV2 and EBHSV in 208 hares displaying lesions compatible with EBHSV at necropsy.
