# Supplementary material for: Large-scale lagovirus disease outbreaks in European brown hares (Lepus europaeus) in France caused by RHDV2 strains spatially shared with rabbits (Oryctolagus cuniculus)
Source: Vet Res. 2017 Oct 28;48:70. doi: 10.1186/s13567-017-0473-y (PMC5660455; doi:10.1186/s13567-017-0473-y)
Supplement: Supplementary file 1 — Additional file 1. Lagovirus positive samples used for the detection and molecular characterization of RHDV2 in hares. The table gives the sample number, the year of collection, the French department number, the genotype and the EMBL/Genbank accession number in nucleotide databases of the RHDV2 VP60 sequence of the lagoviruses characterized in hares (n = 7) and of nine RHDV2 characterized in dead rabbits during the same period of time. *Recombinant RHDV-G1/RHDV2 strains. [file 13567_2017_473_MOESM1_ESM.docx]

| Species | Sample number | Year of collection | French department number | Genotype | RHDV2 accession number (VP60) |
| --- | --- | --- | --- | --- | --- |
| Hare | E13-01 | 2013 | 15 | EBHSV |  |
|  | E13-02 |  | 47 | EBHSV |  |
|  | E13-37 |  | 22 | **RHDV2** | LT168833 |
|  | E14-03 |  | 76 | **RHDV2** | LT168834 |
|  | E14-39 | 2014 | 59 | EBHSV |  |
|  | E14-40 |  | 79 | **RHDV2** + EBHSV | LT168835 |
|  | E14-45* |  | 47 | **RHDV2** | LT168836 |
|  | E14-73 |  | 03 | **RHDV2** | LT549473 |
|  | E14-115* |  | 47 | **RHDV2** | LT168837 |
|  | E15-12* | 2015 | 32 | **RHDV2** | LT168838 |
| Rabbit | 13-22 | 2013 | 46 | **RHDV2** | LT168839 |
|  | 13-69 |  | 30 | **RHDV2** | LT168840 |
|  | 13-71 |  | 29 | **RHDV2** | LT168841 |
|  | 13-100 |  | 53 | **RHDV2** | LT168842 |
|  | 13-122 |  | 47 | **RHDV2** | LT168843 |
|  | 13-165* |  | 32 | **RHDV2** | LT168844 |
|  | 14-02 |  | 84 | **RHDV2** | LT168845 |
|  | 14-59* | 2014 | 47 | **RHDV2** | LT168846 |
|  | 14-114 |  | 39 | **RHDV2** | LT168847 |
