# Supplementary material for: Large-scale lagovirus disease outbreaks in European brown hares (Lepus europaeus) in France caused by RHDV2 strains spatially shared with rabbits (Oryctolagus cuniculus)
Source: Vet Res. 2017 Oct 28;48:70. doi: 10.1186/s13567-017-0473-y (PMC5660455; doi:10.1186/s13567-017-0473-y)
Supplement: Supplementary file 2 — Additional file 2. Lagovirus positive samples used for estimating the proportion of RHDV2 in lagovirus infection in hares. The table gives the sample number, the year of collection, the French department number and the genotype of the lagoviruses characterized during the screening performed to estimate the relative proportion of RHDV2 and EBHSV in 208 hares displaying lesions compatible with EBHSV at necropsy. [file 13567_2017_473_MOESM2_ESM.docx]

| Species | Sample number | Year of collection | French  departement | Genotype |
| --- | --- | --- | --- | --- |
| Hare | E15-01 | 2015 | 47 | EBHSV |
|  | E15-02 |  | 47 | EBHSV |
|  | E15-03 |  | 47 | EBHSV |
|  | E15-05 |  | 47 | EBHSV |
|  | E15-11 |  | 72 | EBHSV |
|  | E15-12 |  | 32 | **RHDV2** |
|  | E15-13 |  | 83 | **RHDV2** |
|  | E15-20 |  | 26 | EBHSV |
|  | E15-23 |  | 45 | EBHSV |
|  | E15-29 |  | 79 | EBHSV |
|  | E15-30 |  | 72 | **RHDV2** |
|  | E15-41 |  | 47 | EBHSV |
|  | E15-42 |  | 81 | EBHSV |
|  | E15-47 |  | 79 | EBHSV |
|  | E15-48 |  | 17 | EBHSV |
|  | E15-55 |  | 29 | EBHSV |
|  | E15-59 |  | 02 | EBHSV |
|  | E15-69 |  | 29 | **RHDV2** |
|  | E15-70 |  | 84 | **RHDV2** |
|  | E15-71 |  | 17 | **RHDV2** |
|  | E15-79 |  | 80 | **RHDV2** |
|  | E15-82 |  | 66 | **RHDV2 +** EBHSV |
|  | E15-83 |  | 11 | EBHSV |
|  | E15-84 |  | 11 | EBHSV |
|  | E15-85 |  | 49 | EBHSV |
|  | E15-86 |  | 12 | EBHSV |
|  | E15-87 |  | 12 | EBHSV |
|  | E15-88 |  | 71 | EBHSV |
|  | E15-89 |  | 12 | EBHSV |
|  | E15-90 |  | 12 | EBHSV |
|  | E15-91 |  | 44 | EBHSV |
|  | E15-93 |  | 54 | EBHSV |
|  | E15-94 |  | 54 | EBHSV |
|  | E15-95 |  | 54 | EBHSV |
|  | E15-96 |  | 54 | EBHSV |
|  | E15-97 |  | 12 | EBHSV |
|  | E15-98 |  | 12 | EBHSV |
|  | E15-99 |  | 54 | EBHSV |
|  | E15-100 |  | 12 | EBHSV |
|  | E15-101 |  | 12 | EBHSV |
|  | E15-102 |  | 59 | EBHSV |
|  | E15-103 |  | 44 | **RHDV2** |
|  | E15-104 |  | 12 | EBHSV |
|  | E15-105 |  | 12 | EBHSV |
|  | E15-107 |  | 81 | EBHSV |
|  | E15-108 |  | 12 | EBHSV |
|  | E15-110 |  | 57 | EBHSV |
|  | E15-111 |  | 57 | EBHSV |
|  | E15-112 |  | 12 | EBHSV |
|  | E15-113 |  | 12 | EBHSV |
|  | E15-115 |  | 39 | EBHSV |
|  | E15-118 |  | 52 | EBHSV |
|  | E15-123 |  | 70 | **RHDV2** |
|  | E15-125 |  | 27 | **RHDV2** |
|  | E15-126 |  | 27 | **RHDV2** |
|  | E15-130 |  | 63 | **RHDV2** |
|  | E15-138 |  | 70 | EBHSV |
|  | E15-144 |  | 81 | **RHDV2** |
|  | E15-145 |  | 81 | **RHDV2** |
|  | E15-146 |  | 81 | **RHDV2** |
|  | E15-147 |  | 12 | EBHSV |
|  | E15-158 |  | 44 | **RHDV2** |
|  | E15-159 |  | 47 | **RHDV2** |
|  | E15-168 |  | 12 | EBHSV |
|  | E15-172 |  | 47 | **RHDV2** |
|  | E15-174 |  | 71 | EBHSV |
|  | E15-175 |  | 71 | **RHDV2** |
|  | E15-182 |  | 47 | **RHDV2** |
|  | E15-183 |  | 44 | **RHDV2** |
|  | E15-187 |  | 54 | EBHSV |
|  | E15-188 |  | 89 | **RHDV2** |
|  | E15-189 |  | 39 | EBHSV |
|  | E15-193 |  | 39 | EBHSV |
|  | E15-194 |  | 07 | **RHDV2** |
|  | E15-195 |  | 07 | **RHDV2** |
|  | E15-196 |  | 44 | EBHSV |
|  | E15-197 |  | 81 | EBHSV |
|  | E15-199 |  | 76 | **RHDV2** |
|  | E15-200 |  | 12 | **RHDV2** |
|  | E15-202 |  | 11 | **RHDV2** |
|  | E15-207 |  | 71 | **RHDV2** |
|  | E15-210 |  | 71 | **RHDV2** |
|  | E15-212 |  | 26 | **RHDV2** |
|  | E15-213 |  | 47 | **RHDV2** |
|  | E15-214 |  | 47 | **RHDV2** |
|  | E16-01 |  | 42 | EBHSV |
|  | E16-02 |  | 80 | EBHSV |
